# Mind–Body Physical Activity Interventions and Stress-Related Physiological Markers in Educational Settings: A Systematic Review and Meta-Analysis

**DOI:** 10.3390/ijerph18010224

**Published:** 2020-12-30

**Authors:** Ildiko Strehli, Ryan D. Burns, Yang Bai, Donna H. Ziegenfuss, Martin E. Block, Timothy A. Brusseau

**Affiliations:** 1 Department of Health and Kinesiology, College of Health, University of Utah, 250 S 1850 E, Salt Lake City, UT 84112, USA; Ildiko.Strehli@utah.edu (I.S.); ryan.d.burns@utah.edu (R.D.B.); yang.bai@utah.edu (Y.B.); donna.ziegenfuss@utah.edu (D.H.Z.); 2 Department of Kinesiology, Curry School of Education, University of Virginia, Charlottesville, VA 22904, USA; meb7u@virginia.edu

**Keywords:** exercise, meta-analysis, Mind–Body, physical activity, stress, yoga

## Abstract

Mind–Body Physical Activity (MBPA) in educational settings is one possible preventive strategy for ameliorating stress-related physiological health parameters. The objectives of this study were to conduct a systematic review of the literature with meta-analyses on the effects of MBPA on stress-related physiological health markers in primary, secondary, and higher education students. In accordance with the Preferred Reporting Items for Systematic Reviews and Meta-Analyses guidelines, the search for peer-reviewed articles published in English was conducted in PubMed, EBSCOhost, PsychInfo, Scopus, and Cochrane Library databases. Criteria for inclusion consisted of empirical studies targeting the student population (primary, secondary, higher education), studies examining the effectiveness of an MBPA intervention, studies including a control or comparison group (pre-test/post-test studies excluded), studies targeting physiological marker outcomes such as heart rate, blood glucose, cortisol, and blood pressure, and finally, studies examining interventions implemented within educational settings. Twenty-six interventions were eligible for the review and quantitative synthesis, which comprised a total of 1625 participants, with 783 students serving within the control/comparison group. There were statistically significant and large pooled effects for MBPA effectiveness for lowering heart rate (Hedges’ g = −1.71, 95% Confidence Interval (CI): −2.43, −0.98), cortisol (Hedges’ g = −1.32, 95% CI: −2.50, −0.16), and systolic and diastolic blood pressure (Hedges’ g = −1.04, 95% CI: −1.53, −0.58). These effects tended to be stronger in older students compared to younger students. Most analyses were characterized as having high heterogeneity and only 10 of the 26 studies were characterized as good quality (38.4%). MBPA interventions may have a positive impact on specific physiological health markers in students, especially in students within higher education. However, higher-quality research is needed in this area.

## 1. Introduction

Ancient forms of Mind–Body activities integrate mental awareness with body movements aimed at improving overall health, while achieving a Mind–Body union with comprehensive health benefits [1,2,3]. Inactivity and stress are exceedingly prevalent in our society and contribute to higher disease prevalence [4,5,6]. Fortunately, regular physical activity (PA) can be of paramount importance to long-term overall health and well-being with protective Mind–Body effects on preventing and reducing disease [6,7,8,9,10]. Given the multidisciplinary Mind–Body preventive benefits of PA and the impact of inactivity on long-term mental and physical well-being, holistic activities, such as Mind–Body therapies, the integration of yoga, tai chi, and qigong originating in Eastern cultures have become popular in Western society, including within educational settings [11,12,13,14,15,16,17,18,19,20,21,22,23,24,25,26,27,28,29,30,31,32,33,34,35,36,37,38,39,40,41,42,43,44,45,46,47].

Mind–Body activities are defined as “whose origins lie outside of the Western culture, typically combines muscle-strengthening, balance training, light-intensity aerobic activity, and flexibility in one package. Some variations of yoga, tai chi, and qigong emphasize relaxation, mindfulness, meditation, or spirituality as well [6]”. PA is any movement performed by the human body that results in skeletal muscle contraction and energy expenditure [6,7,8]. Contributions of a physically active lifestyle to enhancing health and preventing disease throughout the lifespan are well-documented [6,7,8,9,48,49,50,51,52,53,54,55,56]. 

Mind–Body activities, which emphasize mindfulness, were developed over centuries in Eastern culture, and the purposeful practice of their unique styles of efficient movement patterns passed onto future generations to enhance mental, spiritual, and physical health [1,2,3,57]. Mindfulness is generally referred to as non-judgmental present moment awareness and is a desired mental quality [1,2,3,57]. Mindfulness has been researched extensively across disciplines, specifically in psychology [57,58]. A scoping review of 5947 bibliographical records revealed 106 mindfulness-based topics [58]. Mindfulness research can depict five categories, and one of the categories, modality, describes how mindfulness can be practiced and encompasses Mind–Body activities, such as yoga, walking, tai chi, and qigong [58]. Mind–Body activities provide physical and mental health benefits [6]; therefore, “Mind–Body” activities could be a possible solution to physical inactivity. Since PA is any movement that raises energy expenditure above resting levels [6,7,8], we depict that yoga, tai chi, qigong, mindful walking, walking meditation, and physical practice, which was developed in Eastern culture, in other words, origins lie outside of the Western culture as Mind–Body physical activity (MBPA). 

Mindfulness from the mental health domain has been extensively researched and results reveal it to be beneficial for stress reduction [11,24,57,58,59,60,61,62,63,64,65,66,67,68,69,70,71]. For example, a systematic review and meta-analysis of 45 randomized control trials (RCT) revealed that mindfulness mediates the physiological markers of stress, such as cortisol, heart rate, blood pressure, and triglycerides in individuals who practice meditation [72]. In a meta-analysis of 42 RCTs, researchers found that practices including yoga asanas with and without mindfulness-based stress reduction (MBSR) practices appear to improve the hypothalamic–pituitary–adrenal and sympathetic nervous system in a variety of different populations [21]. However, to date, no research has been conducted examining the literature and report the impact of MBPA on stress-related physiological health markers targeting students in educational settings. 

Chronic elevations of physiological health markers, for example, cardiometabolic, stress-related, and inflammatory processes are associated with many chronic diseases, such as cardiovascular disease (CVD) early in life, and regular PA has protective effects [6,7,54,73]. Since it is estimated that about 1 in 3 adults have CVD, and by 2030 it is predicted that 40.5% of the US population will have some form of CVD. Lipid deposits begin to accumulate in the aorta in childhood, as early as 3–5 years old, which leads to the development of fibrous plaque formations in the 20 s MBPAs in education are vital [73,74,75]. 

Educational settings provide numerous opportunities for the intention to introduce structured MBPAs to enhance physical and mental health. Research implementing mindfulness-based interventions for the purpose of treating child and adolescent physical and mental health conditions is encouraging [70,76,77,78]. Results have revealed reduced symptoms of depression, anxiety, withdrawal, craving, chronic pain, and illness [70,76,77,78,79]. Psychological outcomes in educational settings have been extensively researched in terms of applying mindfulness [59,69,80,81,82,83,84,85]. Specifically, a systematic review and meta-analysis of 19 studies conducted from grades 1 to 12 revealed the increasing resilience to stress and cognitive performance [69]. Additionally, a recent meta-analysis of 33 RCTs found significant positive effects in children and adolescents’ cognition and mental health in the outcome categories of mindfulness, executive functioning, attention, depression, anxiety/stress, and negative behaviors [65]. Further, in a recent meta-review of 10 systematic reviews and meta-analysis of psychological school-based interventions to improve well-being and prevent mental illnesses, researchers suggested that mindfulness and yoga are effective practices [59]. 

Given the importance of optimizing MBPAs in education, the primary participant population of interest in this review is students. Students spend years in educational settings where the multidimensional preventive benefits of MBPAs could be established and improve mental and physical health as one package, by focusing on the union of body and mind [1,2,3,6,57]. There is a need to examine further the effects of MBPA interventions aimed at strengthening physiological health markers and preventing disease targeting the student population, e.g., primary (6–12 years old), secondary (13–18 years old), higher education (18 and older), in educational settings. The results of this study will further inform school administrators, health, and physical educators on the importance of integrating MBPA interventions and programs in educational environments to facilitate overall health and well-being targeting students. Therefore, the purpose of this study was to conduct a systematic review and meta-analysis of the literature of MBPAs on stress-related physiological health markers targeting primary, secondary, and higher education students. 

## 2. Materials and Methods

### 2.1. Search Procedures

This systematic review and meta-analysis were conducted following the Preferred Reporting Items for Systematic Reviews and Meta-Analyses (PRISMA) guidelines [86]. The PICOS framework was used, which describes participants (P), interventions (I), comparisons groups (C), outcomes (O), and study design in an educational setting (S) to develop the search strategies and objectives [86]. The search for this study was performed in PubMed, EBSCOhost, PsychInfo, Scopus, Cochrane Library, and researchers used Interlibrary Loan (ILL) and document services as needed to review full texts. Database searches included MBPA interventions published until April 2020. 

The searches were limited to articles published in English and were peer reviewed. Researchers used the following keywords, terms, and combinations appropriate to the specific database; “student”, “youth”, “adolescent”, “child”, “juvenile”, “pediatric”, “Mind–Body”, “yoga”, “mindfulness”, “physical activity”, “movement”, “physical education”, “stress”, “cortisol”, “blood”, and “heart rate” which resulted in 2094 items. Keywords were organized and entered into the database using the PICOS framework and combinations of keywords were entered using the “OR” and “AND” Boolean operators. 

### 2.2. Study Inclusion Criteria

**Figure 1:**
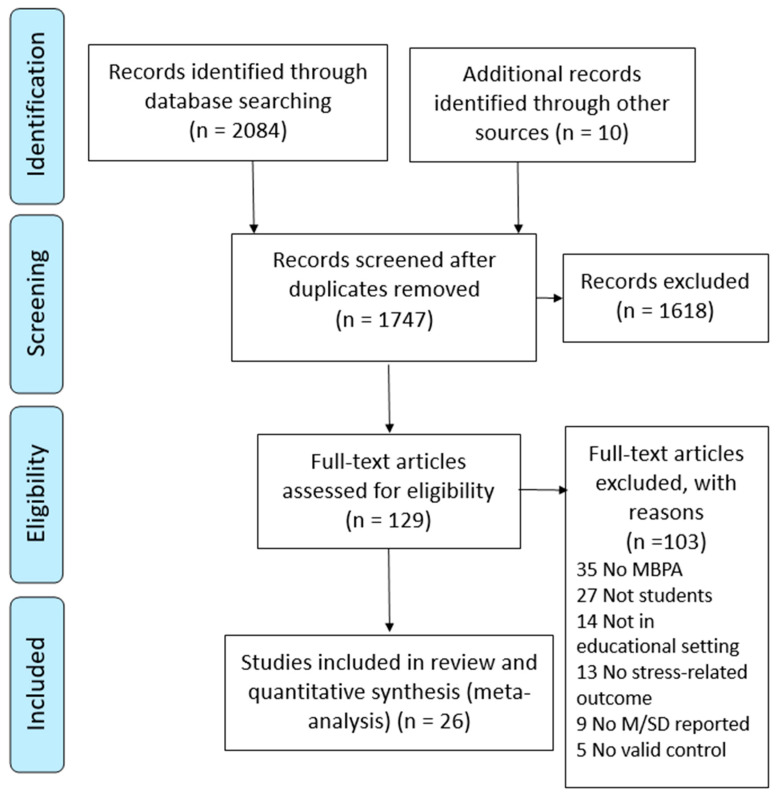
Preferred Reporting Items for Systematic Reviews and Meta-Analyses (PRISMA) flow diagram.

Studies were selected from this initial pool in three steps. The first selection step extracted MBPA intervention studies in educational settings targeting stress-related physiological health markers as outcome variables and targeting primary, secondary, or higher education students. Only experimental and quasi-experimental studies were extracted from the literature; observational studies were excluded. The second step further separated the studies based on whether a control or comparison group was utilized within the research design from studies that used a pre-test/post-test design with no control group. The third and final selection step eliminated those studies where insufficient information was provided within the published manuscript that was needed to calculate the standardized mean differences. The researchers identified a total of 2094 articles. The PRISMA flowchart communicating these procedures is provided in Figure 1. 

### 2.3. Study Quality

Researchers assessed each study’s quality following the National Institutes of Health (NIH) National Heart, Lung, and Blood Institute Study Quality Assessment Tools. The quality of each study was rated as good, fair, or poor. Researchers responded to each study criteria by scoring yes (Y), no (N), and other, which was divided as cannot determine (CD), not applicable (NA), or not reported (NR). After summarizing the scores, each study received a quality rating (good, fair, or poor) [87]. 

### 2.4. Statistical Analysis

The following meta-analyses tested the null hypothesis that the overall effect (weighted pooled Standardized Mean Difference (SMD), Hedges’ g) of MBPA interventions on improving physiological health markers across studies was 0. Using STATA’s “metan” command, DerSimonian and Laird random-effects models were employed with the estimates of between-study heterogeneity taken from the inverse-variance fixed-effect model. Studies were weighted based on inverse variance, which included both within-study and between-study variance (i.e., heterogeneity). Individual study effects and the overall summary effect were reported within a Forest Plot with corresponding 95% Confidence Intervals. Meta-analyses were conducted for each physiological health marker for the total sample and within educational subgroups (i.e., primary, secondary, higher education). Effect estimates were considered small if g < 0.20, medium if g = 0.50, and large if g ≥ 0.80. Between-study heterogeneity across studies was quantitatively assessed using Cochran’s Q test and the I^2^ statistic. Magnitude of between-study heterogeneity was determined small if I^2^ < 50%, moderate if I^2^ = 50–75%, and large if I^2^ > 75%. Publication bias was assessed via visual inspection of Funnel Plots, which displayed the SMDs on the x-axis and the standard errors on the y-axis and statistically assessed using the Egger linear regression test and a Galbraith plot. Funnel Plots were determined asymmetrical, suggesting publication bias, if the intercepts from the Egger regression model and Galbraith plot significantly deviated from 0. The Funnel Plot was obtained using STATA’s “metafunnel” command and the Egger regression parameter estimates with Galbraith plots were obtained using STATA’s “metabias” command. Post hoc sensitivity analyses were conducted, removing a single study from the meta-analyses per iteration to determine if the parameter estimates (Hedges’ g and Egger bias coefficient) would significantly change compared to if that respective study were included in an analysis [88]. Alpha level was set at *p* < 0.05, and all analyses were conducted using STATA v.15.0 statistical software package [89]. 

## 3. Results

### 3.1. Characteristics of the Included Studies

Comprised study characteristics are delineated in Table 1. Twenty-six intervention studies examining the effects of MBPA on stress-related physiological markers, specifically, heart rate, glucose, cortisol, and blood pressure in educational settings were included in this review [15,18,19,26,27,28,29,30,31,34,37,38,42,44,90,91,92,93,94,95,96,97,98,99,100,101]. From the 26 studies, researchers conducted 17 in higher education (65%), 4 in secondary school, 5 in primary school settings, and 9 in India, 8 in the USA, 4 in China, two in South Korea, one in Africa, in Taiwan, and Japan. A total of 1625 students participated in 21 yoga (80.77%), three qigong, one tai chi, and one walking meditation, trained instructor-led interventions with group sample sizes ranging from 8 to 104 783 participants served as the control/comparison group. Of the total number of students, 822 were females; however, three interventions with 340 participants did not report gender [18,34,92]. From the 26 included studies, researchers rated 14 as ‘fair’, 10 as ‘good’, and two as ‘poor’. In the reviewed articles, researchers have randomized the participants in 18 studies (69%) and did not assign participants randomly to intervention and control groups in eight experimental studies [31,32,38,93,95,96,100,101]. They mainly used a convenient sampling technique (i.e., participants only of the researchers’ institute), and one study used multistage cluster sampling [34]. Due to the nature of the MBPA interventions, blinding of researchers and participants was not reviewed as it is difficult.

### 3.2. MBPA Interventions

As demonstrated in Table 1, the majority of the MBPA interventions were yoga-based and guided by certified instructors. Yoga forms were heterogeneous across the reviewed interventions and integrated the eight limbs of practice. For example, from yogic exercises, vinyasa flows, breathing practices, laughter yoga to hatha yoga interventions were used [19,29,37,38,90,98,100,101]. Qigong was the second most common intervention. The three qigong interventions used the “Five Animal” exercise, “Eight Silken” movements, “White Ball”, and “Laughing Qigong” programs [44,92,96]. One study used the 24 forms of simplified Tai Chi Chuan (TCC) [42]. Another intervention used meditation and walking as part of a mental and physical training program [91].

Intervention duration lasted from one 10 min single bout session to six months [18,90]. Generally, participants practiced MBPA sessions from one day to six days weekly, from 15 to 60 min, led by trained instructors for 1 to 24 weeks. Many researchers described the interventions and provided didactic information of used activities, such as asanas, qigong movements, and Tai Chi forms, including instructions for home practice with parents [96,99]. As of the reviewed interventions, one study control group participants, while following their regular routine without MBPA, were required to record their daily physical activities in a log [42]. Six studies used active control groups, such as indoor track training, music, art class, physical education class, general health education [15,37,38,95,97,101]. Seven interventions used inactive control groups, such as seated rest, book reading, seated breathing, and watching a movie [19,30,44,90,91,99,100]. In 12 studies, the control group participants followed their regular daily routine and did not have any MBPA practice during the intervention [18,26,27,28,29,31,34,93,94,96,98].

Of the 26 studies that reported relevant outcome measures of stress-related physiological markers, the most common values were autonomic arousal, such as heart rate (HR), and both systolic (SBP) and diastolic (DBP) blood pressure (BP) parameters. Seventeen interventions reported values of HR [18,26,27,28,30,31,37,42,44,90,91,92,93,94,95,96,97,98]. Researchers measured resting HR in 47% of the included MBPA interventions. Sixteen studies reported values of BP [26,27,28,30,31,34,37,42,44,93,94,95,96,97,98,101]. The steroid stress hormone cortisol was measured in seven studies [19,37,38,44,96,99,100,101]. The other relevant stress-related physiological measure included in this review is blood glucose, which was evaluated in four interventions [15,29,93,98]. To statistically combine the stress-related parameters from the 26 individual MBPA intervention studies, researchers performed meta-analyses by grouping the relevant data. For the meta-analysis objectives, MBPA interventions were analyzed on HR, blood glucose, cortisol, and BP as a total sample, and as subsamples in higher education, secondary school, and primary school settings to achieve the weighted pooled effects of the inclusive sample of 1625 students.

### 3.3. Meta-Analyses

Obtained parameter estimates (weighted pooled effect sizes, Hedges’ g) for each outcome variable are presented in text and within Table 2. Funnel Plots and Galbraith Plots displaying the degree of heterogeneity and publication bias are provided within the supplementary figures for outcomes showing statistical significance using the total sample (Supplementary Figures S1–S6). Sensitivity analyses for each outcome that showed statistically significant pooled effects using the total sample are also presented as supplementary figures (Supplementary Figures S7–S9).

### 3.4. Heart Rate

**Figure 2:**
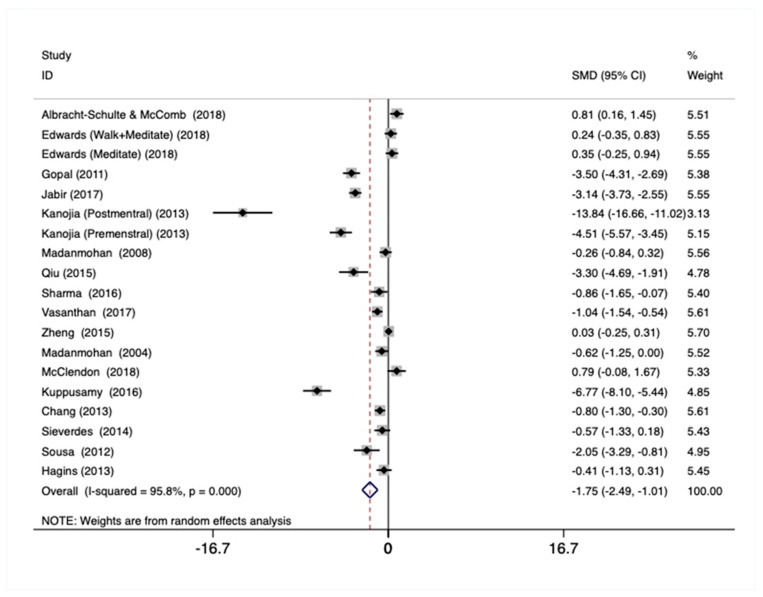
Forest plot showing the weighted pooled effect of Mind–Body physical activity interventions on heart rate.

Total Sample—Using the total sample (N = 19), there was a statistically significant and large pooled effect on heart rate (Hedges’ g = −1.71, *p* = 0.001; Figure 2), which was characterized by large heterogeneity across studies (χ^2^(18) = 412.18, *p* < 0.001, I^2^ = 95.6%). There was also evidence for strong publication bias using Egger regression (bias = −7.85, *p* = 0.014).

Higher Education Subsample—Within the higher education heart rate subsample (n = 12), there was a statistically significant and large pooled effect on heart rate (Hedges’ g = −1.94, *p* < 0.001), which was characterized by high heterogeneity across studies (χ^2^(11) = 319.49, *p* < 0.001, I^2^ = 96.6%). There was also evidence for publication bias using Egger regression (bias = −4.54, *p* = 0.050).

Secondary School Subsample—No statistically significant pooled effects on heart rate were observed within the secondary school subsample (n = 3).

Primary School Subsample—Within the primary school subsample (n = 4), there was a statistically significant and moderate pooled effect on heart rate (Hedges’ g = −0.76, *p* = 0.001), which was characterized by small heterogeneity across studies (χ^2^(3) = 4.66, *p* = 0.199, I^2^ = 35.6%).

### 3.5. Glucose

**Figure 3:**
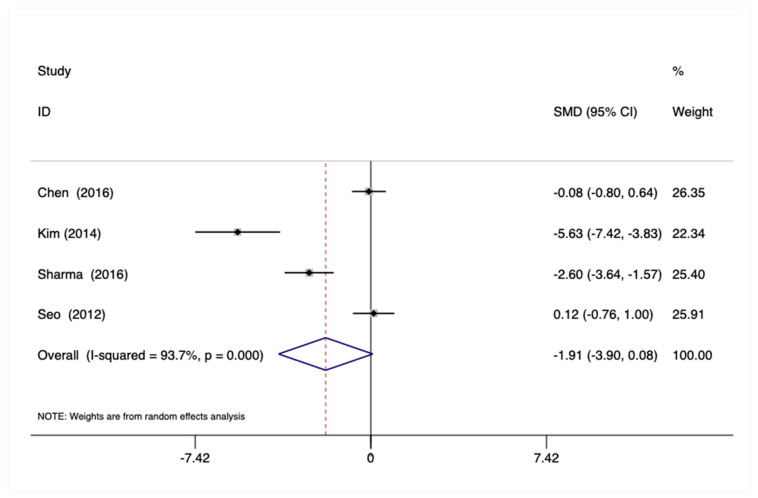
Forest plot showing the weighted pooled effect of Mind–Body physical activity interventions on blood glucose.

Total Sample—No statistically significant pooled effects on glucose were observed using the total sample (Figure 3) or within the educational subsample.

### 3.6. Cortisol

**Figure 4:**
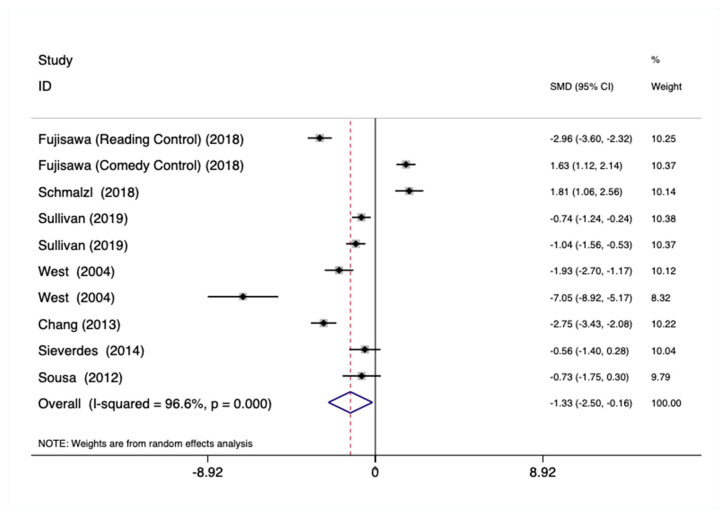
Forest plot showing the weighted pooled effect of Mind–Body physical activity interventions on cortisol.

Total Sample—Using the total sample (N = 10), there was a statistically significant and large pooled effect on cortisol (Hedges’ g = −1.32, *p* = 0.026; Figure 4), which was characterized by large heterogeneity across studies (χ^2^(9) = 261.18, *p* < 0.001, I^2^ = 96.6%). However, there was no evidence for publication bias using Egger regression (bias = −7.01, *p* = 0.243).

Higher Education Sample—No statistically significant pooled effects on cortisol were observed within the higher education subsample (n = 7).

Primary School Sample—No statistically significant pooled effects on cortisol were observed within the primary school subsample (n = 3).

### 3.7. Blood Pressure

**Figure 5:**
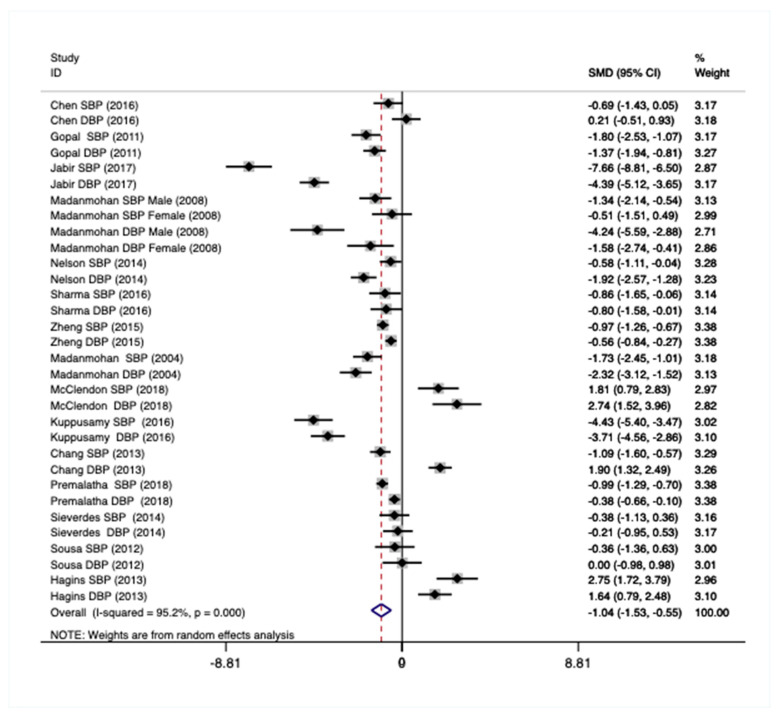
Forest plot showing the weighted pooled effect of Mind–Body physical activity interventions on blood pressure.

Total Sample—Using the total sample (N = 32), there was a statistically significant and large pooled effect on blood pressure (Hedges’ g = −1.04, *p* < 0.001; Figure 5), which was characterized by large heterogeneity across studies (χ^2^(31) = 645.88, *p* < 0.001, I^2^ = 95.2%). However, there was no evidence for publication bias using Egger regression (bias = −1.42, *p* = 0.099). For all analyses, there was no significant difference between systolic and diastolic blood pressure pooled estimates because the 95% Confidence Intervals overlapped.

Higher Education Sample—Within the higher education subsample (n = 16), there was a statistically significant and moderate pooled effect on blood pressure (Hedges’ g = −1.75, *p* < 0.001), which was characterized by high heterogeneity across studies (χ^2^(15) = 267.87, *p* < 0.001, I^2^ = 94.4%). There was also some evidence for publication bias using Egger regression (bias = −4.54, *p* = 0.050).

Secondary School Sample—No statistically significant pooled effects on blood pressure were observed within the secondary school subsample.

Primary School Sample—No statistically significant pooled effects on blood pressure were observed within the primary school subsample.

## 4. Discussion

The purpose of this study was to review and examine the pooled effects of MBPA interventions on stress-related physiological health markers in educational settings and to further our investigation in subsamples of primary, secondary, and higher education students. To our knowledge, this is the first study to review the current evidence on the stress-related physiological benefits of MBPA interventions in students. We identified 26 interventions that were eligible for review, which comprised a total of 1625 participants, with 783 students serving within the control/comparison group. There were statistically significant and large pooled effects for MBPA intervention’s effectiveness for lowering heart rate, cortisol, and systolic and diastolic blood pressure. The subsample analyses revealed significant pooled effects for lowering heart rate and blood pressure in higher education and lowering heart rate in primary school settings. Therefore, the substantial effects are consistent and tend to be stronger in older students than younger students.

Our review identified a diversity of MBPA interventions, durations, and sample sizes. In particular, 80.77% of the interventions were yoga-based, durations lasted from one 10 min single bout session to six months, and group sample sizes ranged from 8 to 104. This diversity of interventions may have influenced the heterogeneity in the present meta-analysis. However, heterogeneity in physical activity interventions is common and can be explained by the diversity in study designs, sample size, assessment methods, and intervention duration. As we defined and conceptualized, MBPA integrates body movements, originated in Eastern culture, with present moment mental awareness and breathing techniques while achieving Mind–Body union. In the PA context, participants may have responded differently to varying intervention durations and intensities. Furthermore, the different ways to measure heart rate may influence the present degree of heterogeneity. In our study, researchers measured heart rate as resting in 47% of the included MBPA interventions. Given that supine and seated positions are common, MBPA intervention researchers may want to consider using supine HR as the baseline measure [37]. Therefore, to diminish clinical heterogeneity, we recommend further investigation since it might be vital to measure resting HR in the rested and seated position, since supine posture might yield an additional relaxing effect [37,90,91]. Additionally, longer duration interventions might yield more favorable neural and physiological responses, and stress-related outcomes, such as improved neural pathways, decreased heart rate, blood pressure, and cortisol. However, 11 different types of single, hour-long yoga sessions with 144 experienced practitioners revealed improved positive emotions, exhaustion, psychological resources, and well-being, measured mainly with ten different subjective instruments [102]. Therefore, we recommend further investigation since it might be vital to measure the effects over time, e.g., investigating shorter timeframes—ten minutes of MBPA interventions compared to longer MBPA interventions on physiological stress-related variables.

While our findings revealed statistically significant and large pooled effects for MBPA intervention effectiveness on heart rate, cortisol, and systolic and diastolic blood pressure outcomes, these effects tended to be stronger in older students than younger students. To adapt to the social, emotional triggers, and physical encounters of life, students can establish holistic self-stress management strategies for growth to achieve overall well-being while in school; therefore, reap the lifetime benefits. Given that “stress refers to that quality of experience, produced through a person–environment transaction, that, through either hyperarousal or under arousal, results in psychological or physiological distress [103]”, MBPA, such as yoga, has revealed encouraging efficacy [5,104]. Cortisol, as a primary stress hormone produced by the sympathetic nervous system (SNS) and the hypothalamic–pituitary–adrenocortical (HPA) axis, aids in controlling blood pressure along with many other neurological and physiological regulatory effects [105]. Therefore, engaging in stress alleviating practices promotes a healthy 24 h cortisol pattern, regulation, secretion, and physical activity is a paramount lifestyle behavior regardless of age and the duration [5,6,7,8,9].

Our results within the primary school subsample indicated that only four studies resulted in statistically significant pooled effects on heart rate, while the higher education subsample of 12 studies resulted in a statistically significant and large pooled effect on heart rate. This finding implies that the positive effects tended to be stronger in older students compared to younger students. Research of MBPA movement practices on children and adolescents is still limited, developing, and determinants are complex [106]. From the PA perspective, children like to play and acquire skills by learning; both contribute to other domains’ developments. The preference for PA changes with age; however, learning new skills and the neural changes (i.e., neuroplasticity) is present across the lifespan [6,106]. From the cardiovascular developmental perspective, HR changes during maturation from childhood to young adult and reduces with age [107]. However, highlighting the potential mechanisms and theories in cardiac autonomic regulation and neurobiological processes is beyond the present project. When children participate in MBPA, which can be considered a light-intensity PA, BP, and HR do not follow the same pattern as in young adults [6,106,107]. Given the practical implication of the effect size and our overall results, we can accept that MBPA applied in educational settings is an essential stress alleviating lifestyle behavior.

When comparing the present results to the previous findings on MPBA interventions, it appears that other reviews support similar outcomes and positive impacts on mental and physical well-being/health [23,24,25,36,40,85,108,109,110,111]. In particular, yoga interventions lowered stress in college students. In a systematic review of 94 yoga interventions in higher education among the emotional health outcomes, 45% of the published studies measured stress [23]. All reviewed articles revealed a positive impact, regardless of the variation in yoga practices [23]. A recent meta-analysis of 23 RCTs measured the effects of meditation, mindfulness, and yoga on anxiety, depression, and stress with 1373 higher education students [24]. Results revealed a moderate effect on stress, measured with a validated questionnaire (i.e., Global Assessment of Recent Stress Scale (GARS), Beck Depression Inventory (BDI), Depression Anxiety Stress Scale−21 (DASS−21), Life Stress Scale for College Students (LSSCS), and State-Trait Anxiety Inventory (STAI)), including many others [24]. In a systematic review of 39 RCTs testing the effects of yoga, 87% of studies reported beneficial impacts on youth health in various settings [110]. In particular, 34 studies improved at least one outcome in psychological/behavioral, physiological/physical, and cognitive domains among youth aged 5–18 [110]. In a diverse 1568 young adult (mean age: 22.0 ± 2.0 years) population-based survey (Eating and Activity over Time) EAT 2018 study, researchers found a high prevalence (43.9%) of exposure to stress and adverse events. However, those who were exposed to adverse events, which were associated with higher stress levels, 12.7% of young adults reported either more or similarly likely to practice yoga, at least 30 min/week [111]. Further, in a systematic review of interventions aimed to reduce perceived stress in graduate nursing students, researchers found that self-care strategies were effective [85]. Furthermore, in 17 systematically reviewed publications that met inclusion criteria, it was found that tai chi was a promising modality for reducing anxiety [40].

When MBPA interventions combined with other mindfulness modalities, for example sitting meditation exercises, it is difficult to assess which physical activity aspect resulted in the positive physiological outcome and was effective. Given that educational settings involve long and physically inactive times, such as sitting, interventions focusing directly on the physical and movement forms of MBPA might reveal the accentuating facets. Strong scientific evidence demonstrates that exposure to high amounts of sedentary behavior increases the risk of mortality, type 2 diabetes, and cardiovascular disease incidence significantly throughout the lifespan [6,7,8,9]. The common goal of MBPA is the integration of mind, body, and spirit (i.e., lifeforce, perception, energy, or vitality) while moving and using the breath, thus improving mental and physical health/well-being by stress reduction. Given the beneficial multidisciplinary background of mindfulness as a mediator, researchers are striving to create interventions, including efficient stress alleviating and preventive adaptation strategies to daily life stressors among students [20,21,45,69,72,112,113,114]. The interaction between physiological and psychological processes will be a particular interest in this field and require multidisciplinary approaches. This study presents essential indicators in MBPA interventions’ role and programs that can be implemented, such as duration and the activities’ dose, and supplements our understanding of practical alternative and complementary solutions to promote and protect students’ well-being. Multicomponent and multilevel MBPA intervention designs in educational settings can tailor individuals’ stress management efforts for optimal well-being. Moreover, our review and meta-analysis are the first to report the potential stress alleviating effects of MBPA interventions in educational settings.

It is imperative to consider that this study’s major limitation is that many of the included interventions do not have a physically active control/comparison group and do not include a follow-up period. These methodological limitations of the reviewed interventions might influence heterogeneity. Given the infancy of this field, our meta-analysis has presented a diversity of intervention durations and sample sizes, which might affect the heterogeneity of the present study. Most analyses were characterized as having high between-study heterogeneity, and 38.4% of the studies were characterized as good quality. Therefore, MBPA interventions may have a positive impact on the analyzed physiological markers in students. This vital field would benefit from a more robust methodology, such as testing hypothesized moderators of intervention effects and mediating mechanisms, incorporating random assignments, appropriate control/comparison groups, follow-up time points, and the use of objective measures for MBPA and physiological stress assessment. Stress-related/physiological health markers are critical to understanding mechanisms by which MBPA is affecting health, extending beyond the motor domain by differentiating the physically active from inactive components.

## 5. Conclusions

Stress is a societal burden; there is evidence that MBPA ameliorates stress, and relating research is growing. This review and meta-analysis are the first to demonstrate that MBPA interventions may positively impact specific physiological markers in students, especially within higher education. Young adults need to establish lifelong stress management and physical activity habits to promote well-being. Future research should include more rigorous methodology, including more studies with sufficient statistical power, study randomization, and appropriate control groups. Incorporation of MBPA within educational settings can improve the physical, mental, and emotional health of youth and young adults.

## Figures and Tables

**Table 1:** Summary of Characteristics of the Included Interventions.

Reference Location	Design	Setting	Participants	MBPA Mindfulness-Based Physical Activity	Physiological Health Markers Relevant Outcome Measures	Intervention	Study Quality
Albracht-Schulte & McComb, 2018 USA	2-way randomized repeated-measures crossover clinical trial	HE University	40 healthy, female college students Mean Age = 20.18 SD = 1.97 IG = 20 CG = 20	YogaFit Beth Shaw’s Vinyasa Flow Series	Heart Rate HR (bpm) time-and frequency-domain measures of HRV, and state anxiety were assessed baseline, post- condition, and post-exposure to emotional stimuli	IG: one 30 min session of YogaFit CG: time-matched seated rest condition on separate days; after each condition, participants viewed 30 min of emotional picture stimuli	Good Trial registration: Retro- spectively registered 2/16/2018, clinical-trials.gov, Identifier: NCT 03458702
Edwards et al., 2018 USA	RCT	HE University	N = 110 Mean Age = 21.4 SD = 2.4 Male 27% IG: walk n = 22, meditation n = 22, walk then meditation n = 22, meditation then walk n = 22 CG: n = 22	10-min yoga instructor guided mindfulness meditation, cues focused on breath/body present moment awareness through deep breathing exercises and a full-body scan	HR (bpm) at 8 time points: baseline, 5 min into the walk, 9 min into the walk, 5 min into the meditation, 9 min into the meditation, 3 min post walk/meditation Resting HR State anxiety Rating of Perceived Exertion RPE Borg 6–20	mental and physical (MAP) training, 10 min single bout IG: walk, meditate, walk then meditate, meditate then walk CG: sit inactive control	Good
Gopal et al., 2011 India	RCT	HE Medical college	N = 60 Female medical students 17–20 years old IG n = 30 CG n = 30	Yogic techniques Yogic prayer 2 min Sukshma Vyayam, micro exercises (6 min) Sthula Vyayama, macro exercises (4 min) Asanas, postures (12 min) Pranayama (4 min) Dhyana, meditation (5 min)	HR (bpm) baseline BP (SBP, DBP) Respiratory rate as physiological parameters with examination stress Biochemical serum parameters	IG: 12 weeks 35 min daily integrated yoga CG: no yoga, normal daily routine	Good
Jabir, Sadananda & Das, 2017 India	RCT	HE Medical college	N = 100 Mean Age = 18.26 SD = 0.63 Male 42% IG n = 50 CG n = 50	Relaxing Asana, Balasana, Child Pose guided by a yoga therapist	Cardiac parameters HR baseline (bpm) BP (SBP, DBP) Mean arterial pressure Rate pressure product	IG: 90 days 10 min CG: without Asanas and yoga practice	Good
Kanojia et al., 2013 India	RCT	HE Medical College	N = 50 Mean Age = 18.6 SD = 1.08 Female IG n = 25 CG n = 25	Integrated yoga Guided by qualified yoga trainer Yogic prayer (2 min) Sukshma Vyayam, micro exercises (5 min) Sthula Vyayama, macro exercises (5 min) Pranayama (3 min) Asanas, postures (20 min) Dhyana, meditation (5 min)	HR resting (bpm) BP (SBP, DBP) as autonomic parameters, parasympathetic reactivity tests, sympathetic reactivity tests	IG: integrated yoga practice 40 min, 6 days/week for 3 menstrual cycles CG: without yoga practice	Good
Madanmohan et al., 2008 India	Quasi-experimental	HE	N = 46 17–20 years old male = 30 female = 16 students IG n = 23 CG n = 23	Asanas, pranayams, and shavasan trained yoga instructor	HR resting (bpm) BP (SBP, DBP) maximum inspiratory and expiratory pressure	IG: 90 min yoga 6 days/week for 6 weeks CG: without yoga practice	Fair
Qiu, Wang & Qin, 2015 China	RCT	HE Sports college	N = 30 21 and 22 years old IG A n = 10 IG B n = 10 CG n = 10	Qigong IG A: Five-animal Exercise and IG B: Eight Silken Movements	HR baseline (bpm) Cardiac functions	IG (A and B): 5 × 60 min for 20 weeks CG: without qigong practice	Fair
Sharma et al., 2016, India	Quasi-experimental	HE Nursing college	N = 20 Nursing students, age from 17 to 21 IG n = 10 CG n = 10	Yoga kriya	HR baseline (bpm) Blood Glucose BP (SBP, DBP)	IG: 30 days yoga kriya for 45 min for 6 days in a week CG: without yoga practice	Poor
Vasanthan et al., 2017 India	RCT (wait listed as a control)	HE Centre for Yoga Therapy Education and Research	N = 109 healthy volunteers age between 20 and 25 years IG1: n = 38 IG2: n = 38 CG n = 33	IG1: received pranayam: pranav, savitri, nadi shuddhi and chandra nadi, IG2: received asan: pawanmuktasana, balasan, dharmikasan and shavasan	HR baseline (bpm) cardiovascular responses	25 min/day for 6 days/week for 6 months IG1: received relaxing pranayam IG2: received relaxing asan, and CG: was wait listed	Fair
Zheng et al., 2015 China	Two-arm, randomized, parallel controlled trial	HE Fujian University of Traditional Chinese Medicine	N = 198 Mean Age = 20.6 SD = 1.1 Female = 133 (67.2%) IG n = 95 CG n = 103	Tai Chi Chuan (TCC) 24 forms of simplified TCC	HR resting (bpm) BP (SBP, DBP) cardio-pulmonary function	IG: 60 min TCC sessions, 5 days per week for 12 weeks CG: activities as usual, daily diary of PA was recorded Follow-up period: 12 weeks with required PA log	Good
Madanmohan et al., 2004 India	Quasi-experimental	Secondary School	N = 43 Mean Age = 16.1 SD = 0.2 Male = 22 IG n = 26 CG n = 17	Yoga, shavasan training	HR resting (bpm) BP (SBP, DBP)	IG: 15 min daily training, 4 days a week for 6 weeks CG: without yoga training	Poor
McClendon & Scott 2018 Africa	Quasi-experimental	Secondary After school program	N = 22 Mean Age = 14.1 SD = 0.83 Male = 5 IG n = 11 CG n = 11	Yoga (pre-existing after school program)	HR resting (bpm) BP (SBP, DBP)	IG: 4 x week for 3 weeks CG: indoor track training	Fair
Kuppusamy et al., 2016 India	RCT	Secondary School	N = 60 Mean Age = 14.56 SD = 2.01 Male = 38 Female = 22 IG n = 30 CG n = 30	Bhramari pranayama	HR resting (bpm) BP (SBP, DBP)	IG: one 45 min guided Bhramari pranayama CG: normal breathing	Good
Chang et al., 2013 Taiwan	Randomized prospective experimental control group	Primary School	N = 67 Mean Age & SD of 7th graders not specified Male = 34 Female = 33 IG n = 34 CG n = 33	Laughing Qigong Program (LQP)	HR baseline (bpm) Cortisol BP (SBP, DBP)	8 weeks 45 min “study-hall” sessions IG: LQP CG: book reading or homework	Fair
Sieverdes et al., 2014, USA	RCT	Primary School	N = 28 Mean Age = 12.3 SD = 0.4 Girls = 57% IG n = 14 CG n = 14	Hatha yoga program (HYP)	HR resting (bpm) Cortisol BP (SBP, DBP)	12 weeks 90 min (5 sessions biweekly) IG: HYP CG: attention control (AC) music or art class	Fair
Sousa et al., 2012 China	Prospective wait list controlled	Primary School	N = 16 Mean Age = 11.5 SD = 0.7 Male = 3 IG n = 8 CG n = 8	Qigong “White Ball”	HR baseline (bpm) Cortisol BP (SBP, DBP)	7 weeks 2 × 30 min guided and daily home practice with parents & record activities IG: qigong CG: without qigong training	Fair
Hagins, Haden & Daly, 2013 USA	RCT blinded evaluators	Primary School	N = 30 Mean Age = 10.75 SD = 0.45 Male 17 Female = 13 IG n = 15 CG n = 15	Yoga	HR baseline (bpm) BP (SBP, DBP) on stress reactivity	15 weeks 3 × 50 min IG: yoga CG: PE class	Good
Chen et al., 2016 China	RCT single-arm parallel	HE	N = 30 healthy females between 18 and 25 Female = 30 IG n = 15 CG n = 15	Hatha yoga	Blood Glucose BP (SBP, DBP)	8 weeks 2 × 60 min IG: Hatha yoga CG: without yoga training	Good
Kim, 2014 Republic of Korea	RCT	HE	N = 27 female nursing students Mean Age = 21.0 SD = 0.2 IG n = 12 CG n = 15	Yogic exercises: surya namaskara, shavasana, and yoga nidra	Blood Glucose on life stress	12 weeks 1 × 60 min IG: yoga CG: without yoga training	Fair
Seo et al., 2012 South Korea	RCT	Secondary School	N = 20 obese boys Mean Age = 14.7 SD = 0.5 Male = 20 IG n = 10 CG n = 10	Yoga Asana training	Blood Glucose	8 weeks 3 × 60 min IG: yoga asana CG: general health education	Fair
Fujisawa et al., 2018 Japan	RCT	HE Medical College	N = 120 Mean Age = 24.4 SD = 3.8 Male = 76 Female = 44 IG n = 40 CG n = 40 CG n = 40	Laughter yoga (LY)	Cortisol	IG: one 30 min LY CG: 30 min comedy movie CG: 30 min quiet reading without humor	Good
Schmalzl et al., 2018 USA	RC pilot	HE University	N = 40 Mean Age = 24.91 SD = 5.99 Male = 17 Female = 23 IG n = 22 CG n = 18	Yoga: movement Ashtanga Vinyasa; seated breathing exercises: ujjayi breath and variations	Cortisol	8 weeks 1 × 45 min instructor led 6 × 20 min home program IG: yoga movement CG: seated breathing	Fair
Sullivan et al., 2019 USA	Quasi-experimental within subject	HE University graduate and undergraduate women	N = 33 Female Mean Age = 21 SD = 2	Yoga: stretch and power	Cortisol	1 × 60 min IG: stretch yoga, IG: power yoga, CG: watching a movie	Fair
West et al., 2004 USA	Quasi-experimental	HE Undergraduate	N = 69 Students enrolled in African dance, Hatha yoga, and biology classes Mean Age = 19 With range 17 to 24 Male = 22 Female = 47 IG n = 21 IG n = 18 CG n = 30	Hatha yoga	Cortisol	3 weeks Participants enrolled in IG: African dance, IG: Hatha yoga CG: biology class	Fair
Nelson et al., 2014 USA	Quasi-experimental	HE University	N = 56 Mean Age = 28.24 SD = 10.64 Male = 30 Female = 26 IG n = 25 CG n = 31	Hatha yoga guided by trained instructor	BP (SBP, DBP)	16 weeks 2 × 45 min IG: introductory Hatha yoga CG: lecture	Fair
Premalatha et al., 2018 India	RCT	Primary School	N = 201 Children 10 to 12 years old IG n = 104 CG n = 97	Yoga guided by trained instructor	BP (SBP, DBP)	6 months 5 days/week 60 min IG: 45 min physical exercise and 15 min yoga asana practice CG: daily routine	Fair

Abbreviations: HE = Higher Education, IG = intervention group, CG = control group, SD = standard deviation, RCT = randomized control trial, HR = heart rate, BP = blood pressure, SBP = systolic blood pressure, DBP = diastolic blood pressure.

**Table 2:** Random-effects meta-analysis effect sizes across outcomes and education subsamples.

Outcome	Sample	Hedges’ g (95% CI)	*p*-Value	I^2^ Statistic
Heart Rate	Total Sample (N = 19)	**−1.71 ^†^** **(−2.43, −0.98)**	<0.001	95.6%
	Higher Education (n = 12)	**−1.94 ^†^** **(−2.93, −0.96)**	<0.001	96.6%
	Secondary School (n = 3)	−2.13 (−5.54, 1.28)	0.222	97.7%
	Primary School (n = 4)	**−0.76 ^†^** **(−1.22, −0.30)**	0.001	35.6%
Blood Glucose	Total Sample (N = 4)	−1.91 (−3.90, 0.08)	0.060	93.7%
Cortisol	Total Sample (N = 10)	**−1.32 ^†^** **(−2.50, −0.16)**	0.026	96.6%
	Higher Education (n = 7)	−1.33 (−2.83, 0.17)	0.082	97.3%
	Primary Schools (n= 3)	−1.37 (−2.89, 0.15)	0.077	89.9%
Blood Pressure	Total (N = 32)	**−1.04 ^†^** **(−1.53, −0.55)**	<0.001	95.2%
	Higher Education (n = 16)	**−1.75 ^†^** **(−2.40, −1.09)**	<0.001	94.4%
	Secondary School (n = 6)	−1.30 (−3.31, 0.72)	0.207	96.7%
	Primary School (n = 10)	0.24 (−0.45, 0.94)	0.490	93.6%

Note: ^†^ and bold denotes statistical significance, *p* < 0.05.

## Data Availability

Not applicable.

## Supplementary Materials

Supplementary tables and figures are available online at https://www.mdpi.com/1660-4601/18/1/224/s1. Figure S1: Funnel plot showing standard error against the standardized mean difference for heart rate, Figure S2: Funnel plot showing standard error against the standardized mean difference for cortisol, Figure S3: Funnel plot showing standard error against the standardized mean difference for blood pressure, Figure S4: Galbraith plot showing standard normal deviate against study precision for heart rate, Figure S5: Galbraith plot showing standard normal deviate against study precision for cortisol, Figure S6: Galbraith plot showing standard normal deviate against study precision for blood pressure, Figure S7: Sensitivity analysis showing how omitting a single study changes the parameter estimate for heart rate, Figure S8: Sensitivity analysis showing how omitting a single study changes the parameter estimate for cortisol, Figure S9: Sensitivity analysis showing how omitting a single study changes the parameter estimate for blood pressure.
