# Supplementary material for: Mind–Body Physical Activity Interventions and Stress-Related Physiological Markers in Educational Settings: A Systematic Review and Meta-Analysis
Source: Int J Environ Res Public Health. 2020 Dec 30;18(1):224. doi: 10.3390/ijerph18010224 (PMC7795448; doi:10.3390/ijerph18010224)
Supplement: Supplementary file 1 [file ijerph-18-00224-s001.pdf]

Supplementary Figures

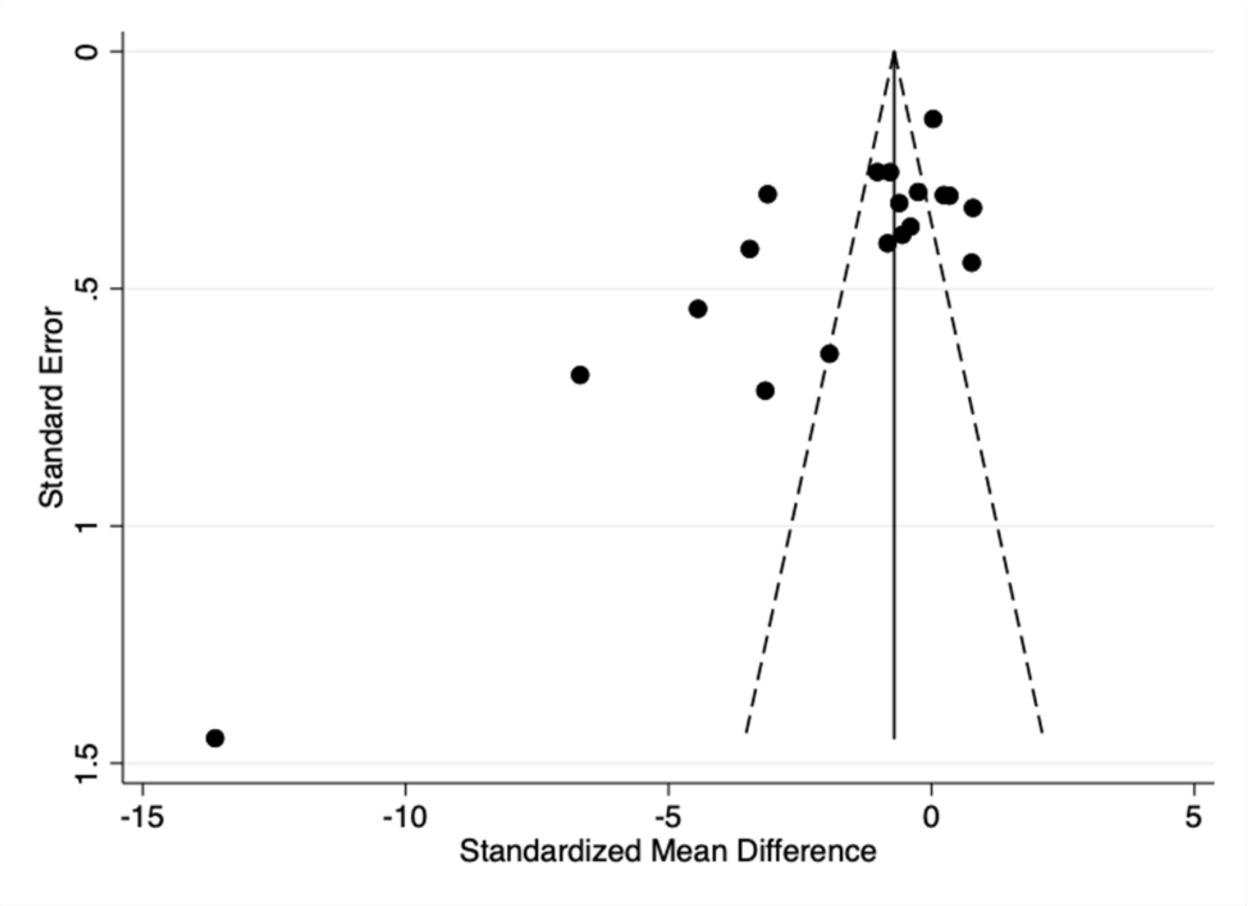

Figure S1. Funnel plot showing standard error against the standardized mean difference for heart rate.

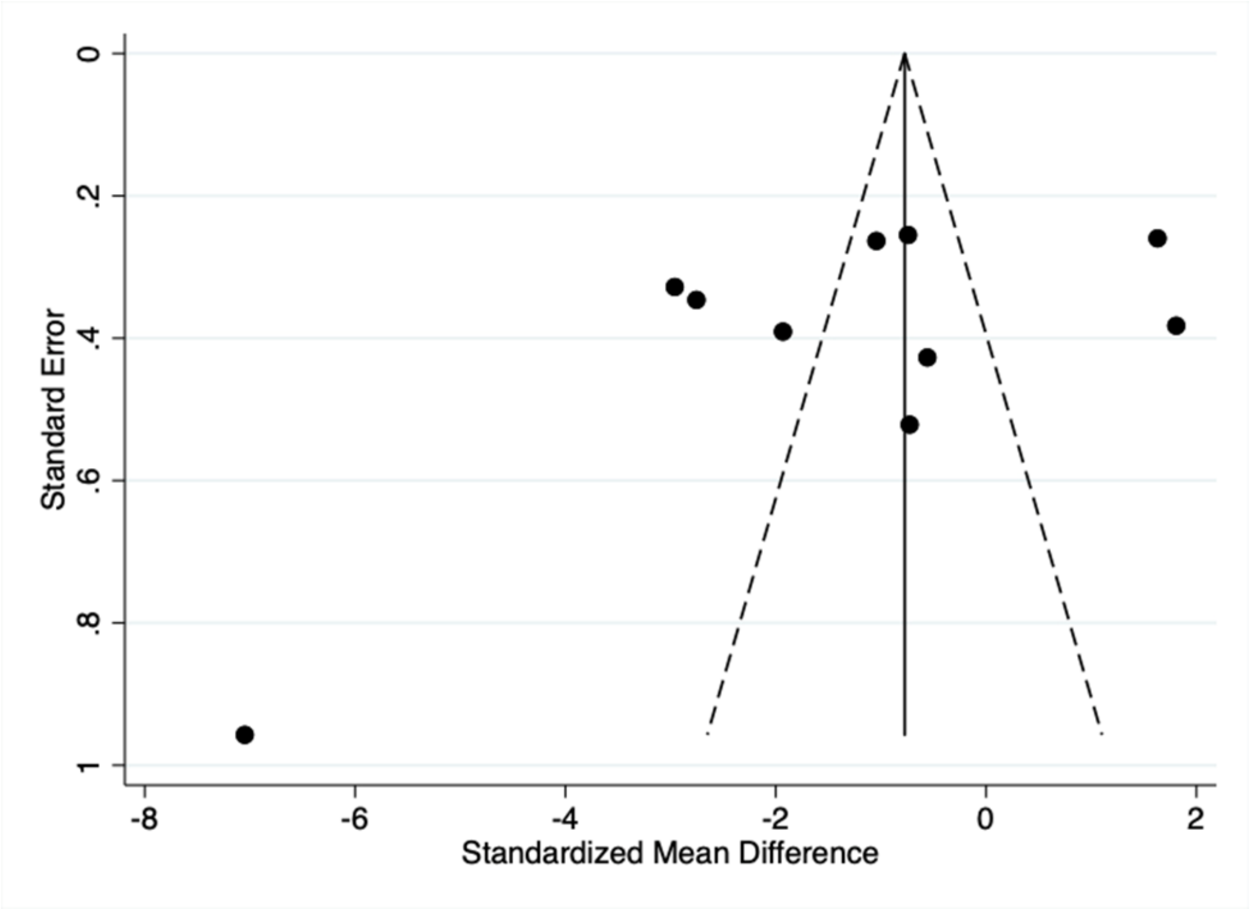

Figure S2. Funnel plot showing standard error against the standardized mean difference for cortisol.

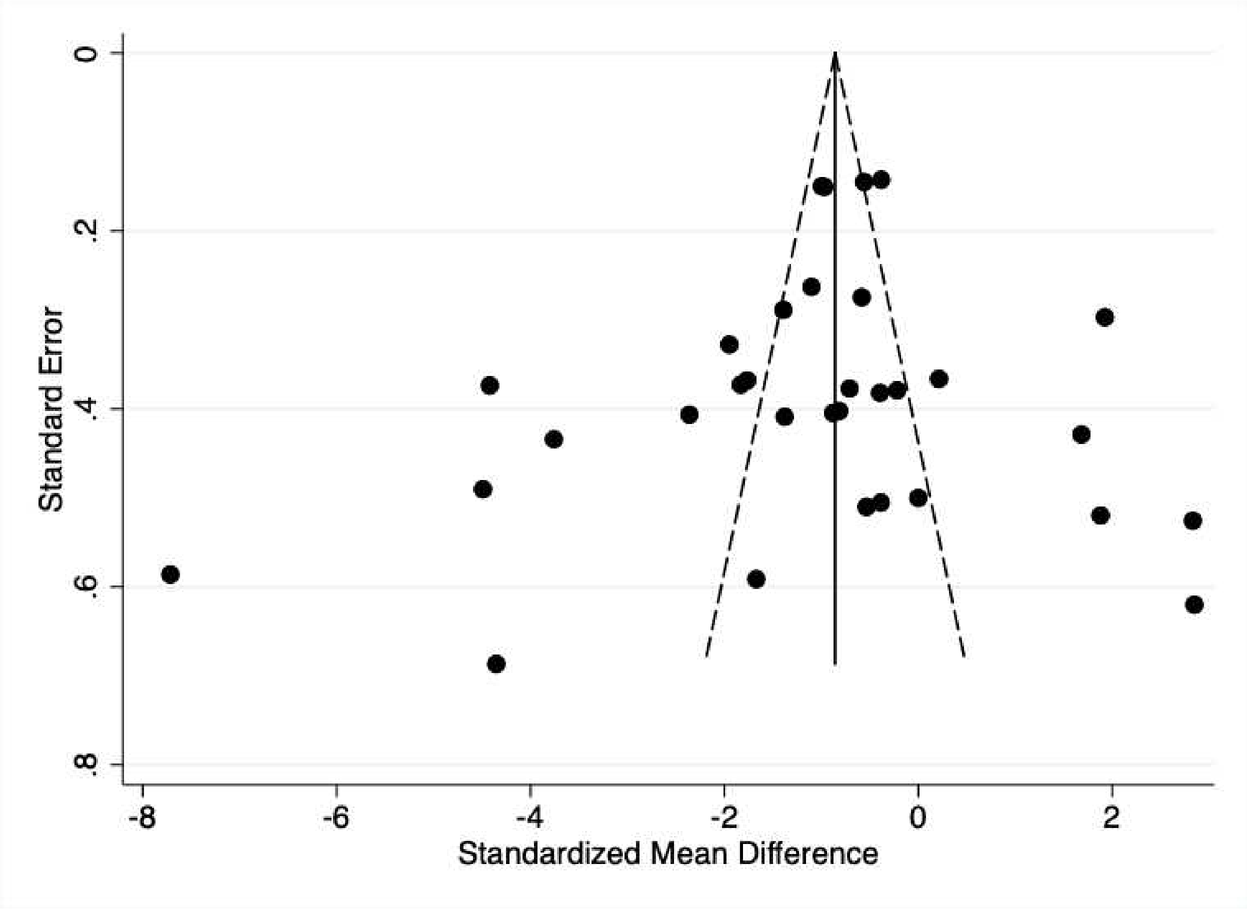

Figure S3. Funnel plot showing standard error against the standardized mean difference for blood pressure.

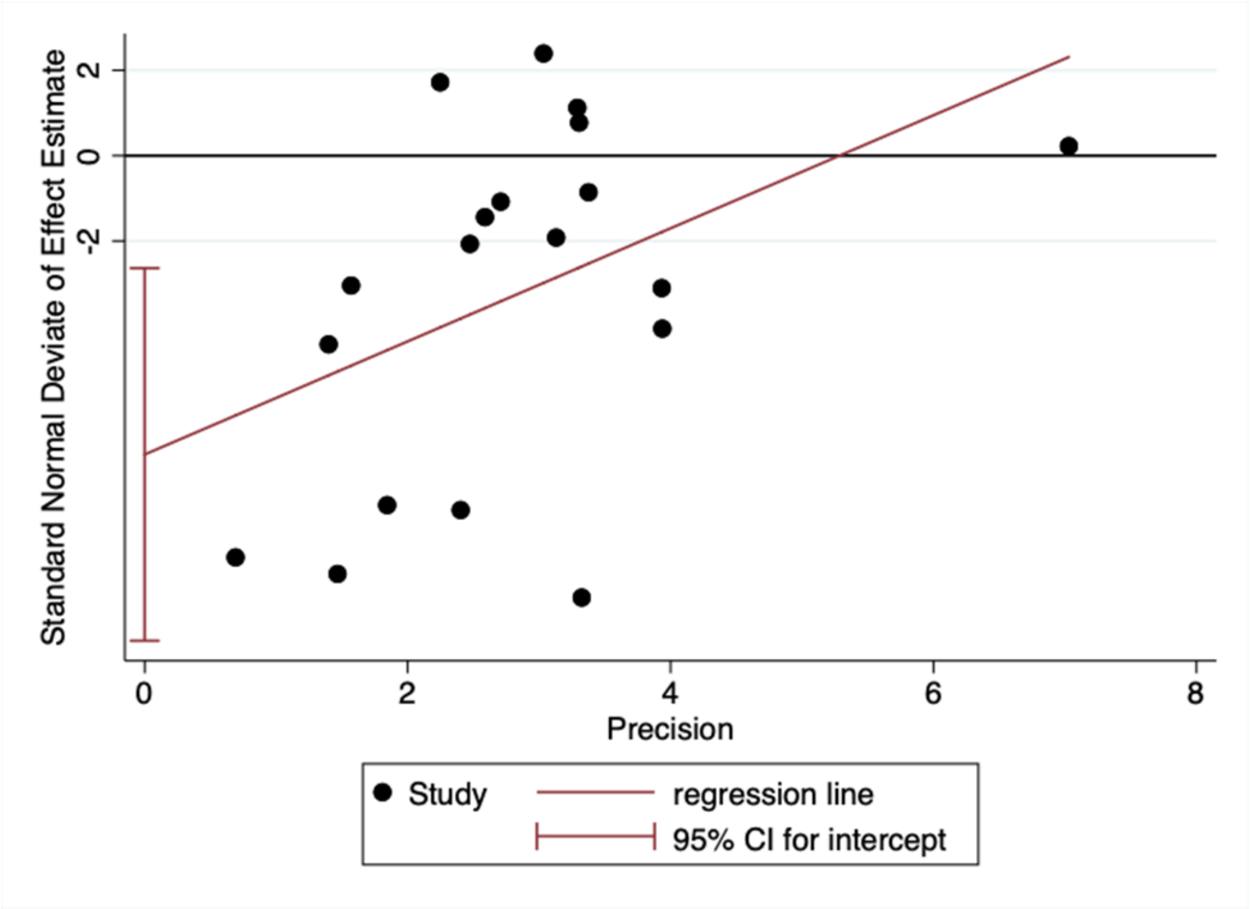

Figure S4. Galbraith plot showing standard normal deviate against study precision for heart rate.

Note: 95% Confidence Interval not crossing 0 x-axis reference line indicates presence of publication bias.

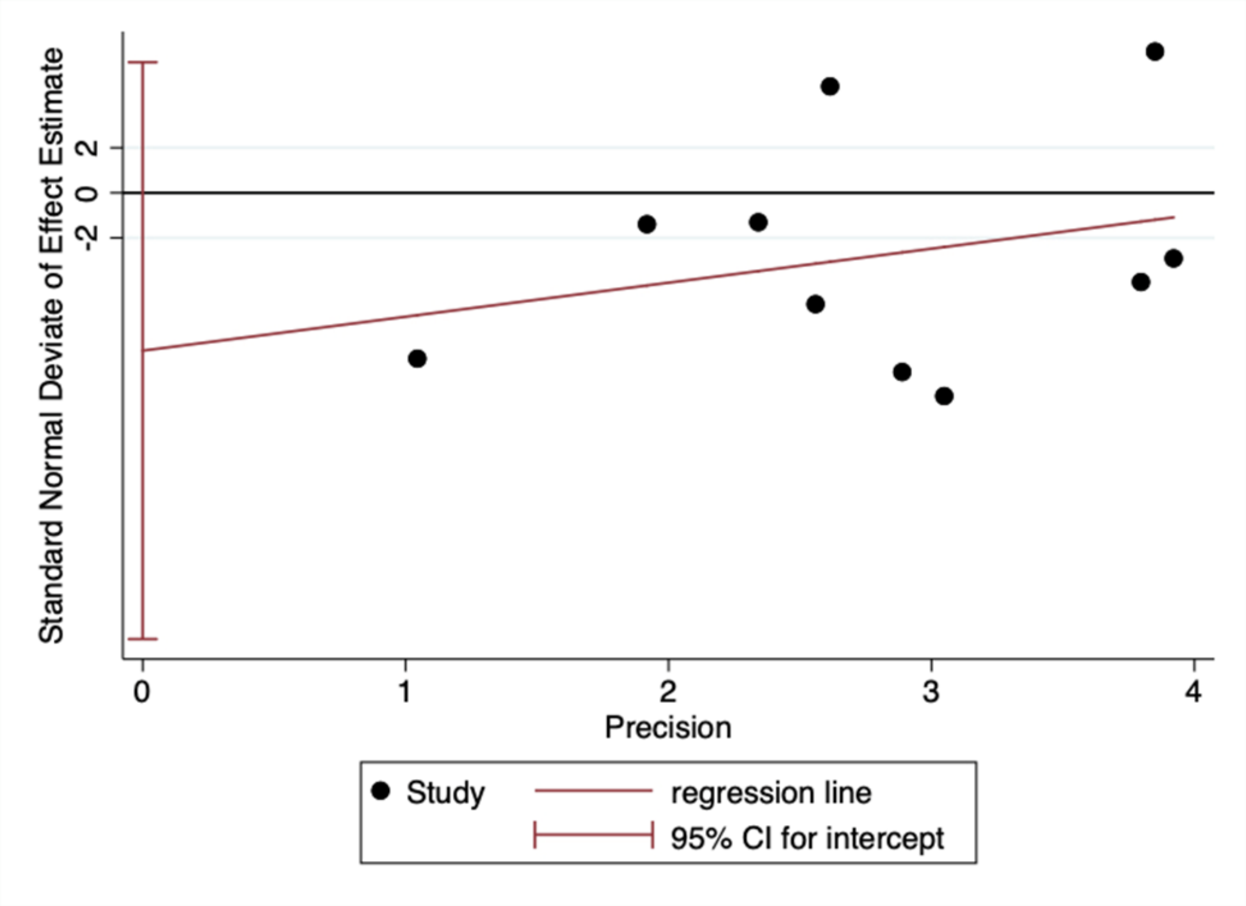

Figure S5. Galbraith plot showing standard normal deviate against study precision for cortisol.

Note: 95% Confidence Interval crossing 0 x-axis reference line indicates no publication bias.

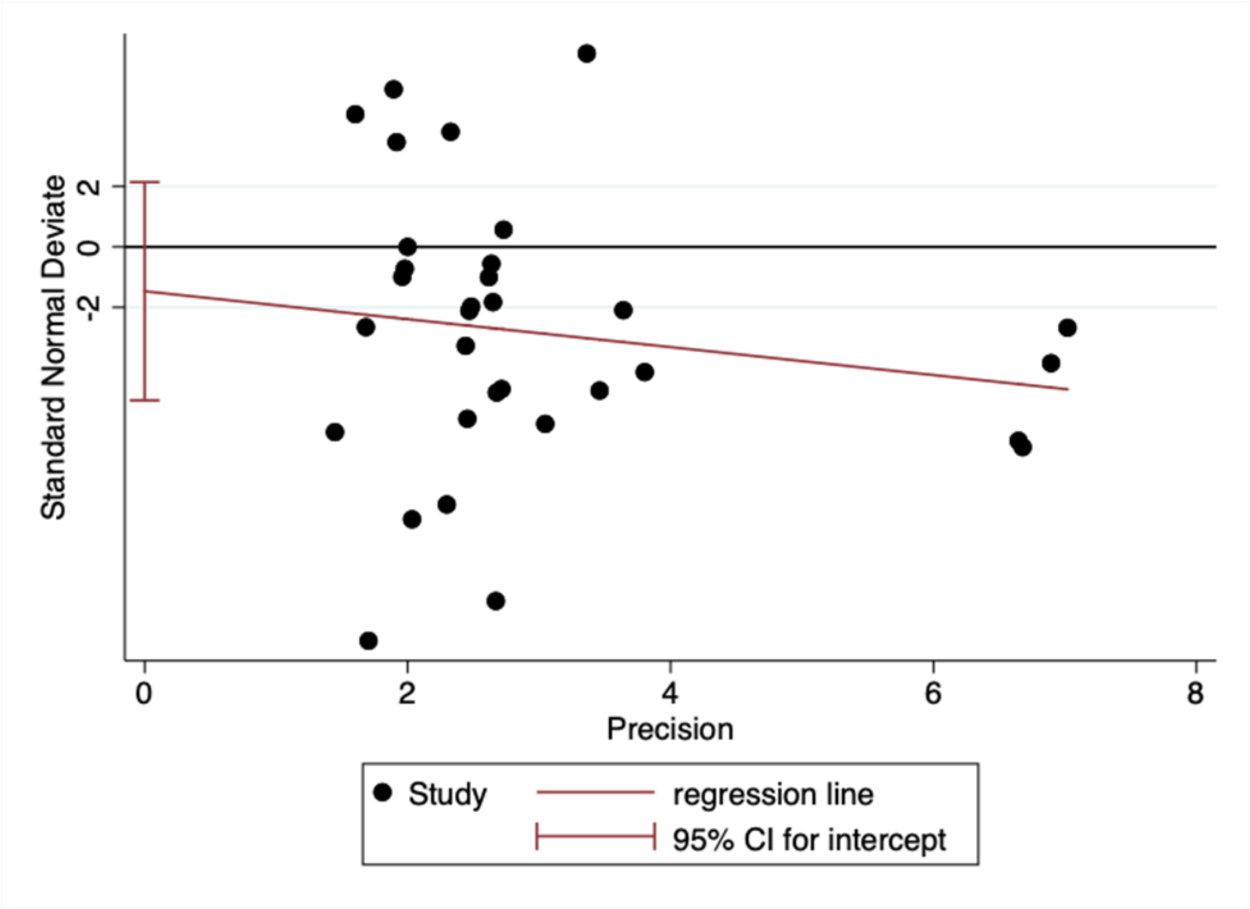

Figure S6. Galbraith plot showing standard normal deviate against study precision for blood pressure.

Note: 95% Confidence Interval crossing 0 x-axis reference line indicates no publication bias.

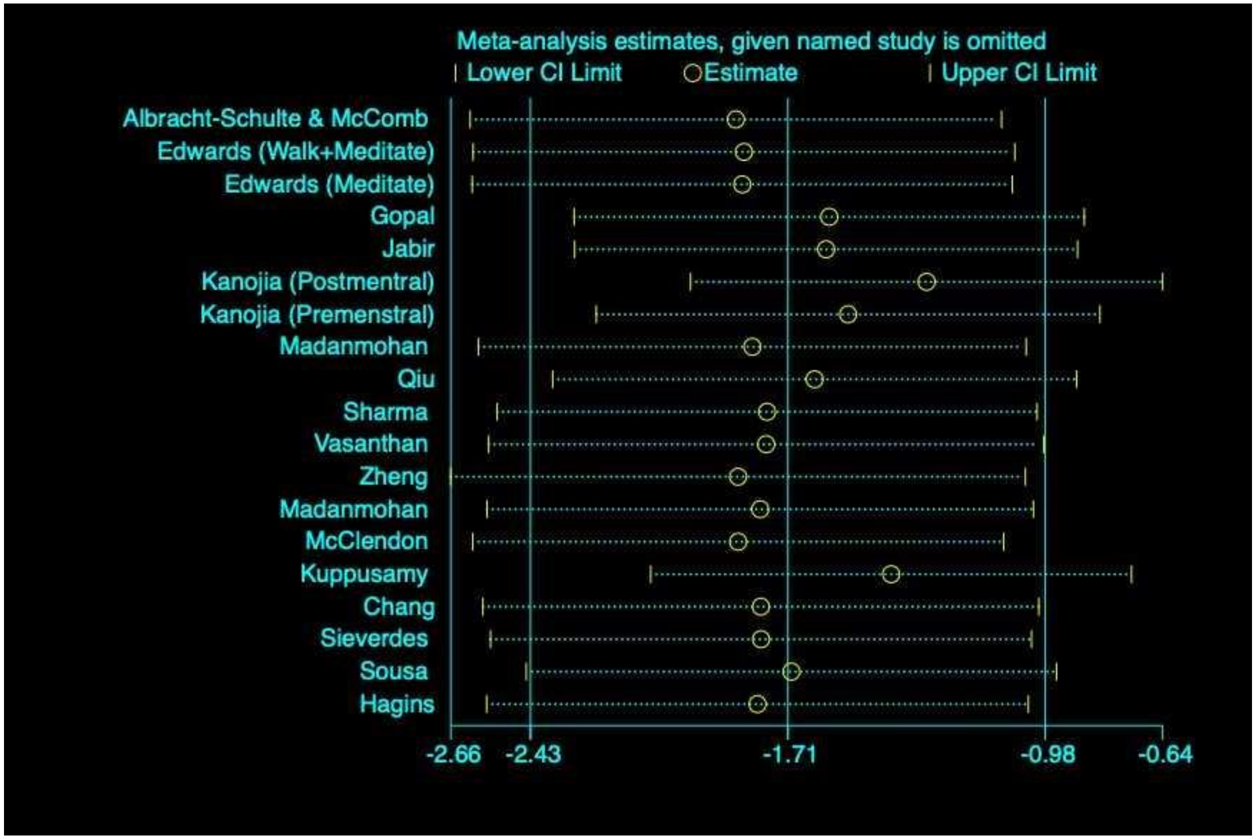

Figure S7. Sensitivity analysis showing how omitting a single study changes the parameter estimate for heart rate.

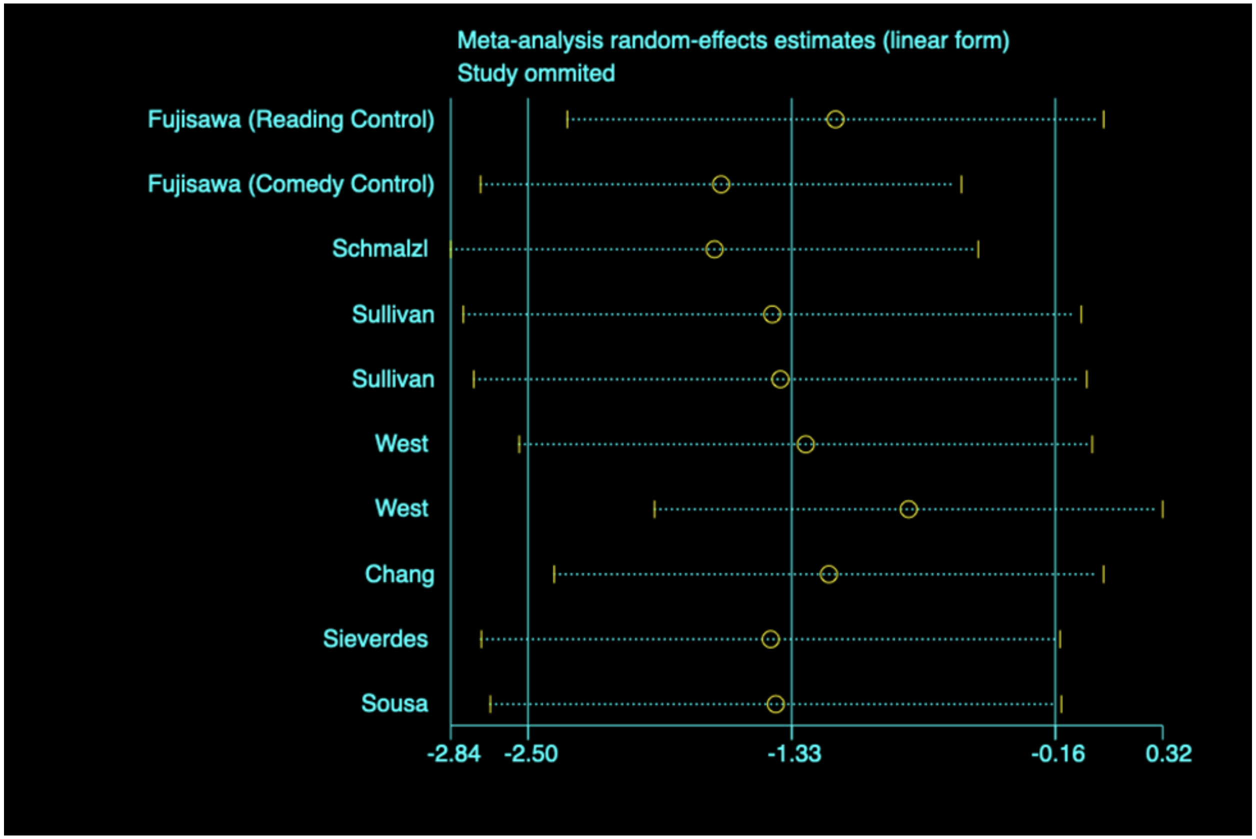

Figure S8. Sensitivity analysis showing how omitting a single study changes the parameter estimate for cortisol.

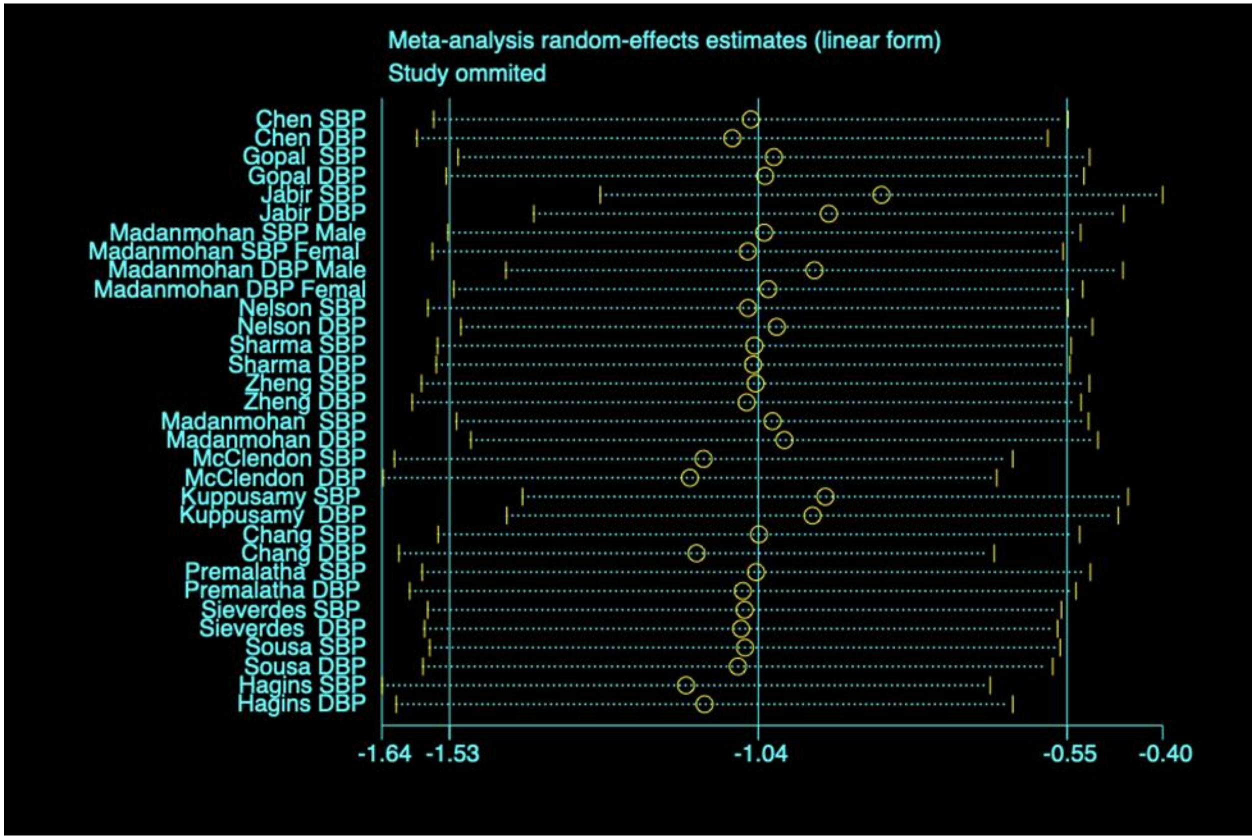

Figure S9. Sensitivity analysis showing how omitting a single study changes the parameter estimate for blood pressure.

SUPPLEMENTARY MATERIAL

Database Search Strategies to Identify Studies

Keywords were organized and entered into the database using PICOS framework (student population (P), must be a mind-body physical activity, yoga, tai chi, qigong, walking, intervention (I), must have a control or comparison group, pre-posttest, follow up period (C), the outcome measures must be physiological health markers, and potential objectively measured PA (accelerometer or pedometer) (O), educational setting, study design (S), and combinations of keywords were entered using the “OR” and “AND” Boolean operators.

The searches were limited to articles published in English; study type: published as a peer-reviewed original article. The following keywords, terms, and combinations appropriate to the specific database were used; “student”, “youth”, “adolescent”, “child”, “juvenile”, “pediatric”, “mind-body”, “yoga”, “qigong”, “tai chi”, “mindfulness”, “physical activity”, “movement”, “physical education”, “stress”, “cortisol”, “blood sugar”, “glucose”, “blood pressure”, “heart rate”, “intervention”, “education”, “primary”, “secondary”, “university”, “tertiary”, “graduate”, “college.” Studies were selected from this initial pool.

Search Terms

PubMed (Medline, PubMed.gov) database internet search was performed using the keywords (student OR youth OR adolescen* OR child OR children* OR juvenile OR pediatric OR paediatric OR “young adult” [All Fields])) AND (mind-body OR yoga OR qigong OR “tai chi” OR mindfulness OR mindful OR walking OR "physical activity" OR movement OR "physical education"[All Fields])) AND (stress OR cortisol OR glucocortic* OR “blood pressure” OR “heart rate” OR “blood sugar” OR glucose) AND (intervention OR experiment OR education OR primary OR secondary OR university OR tertiary OR graduate OR college[Title]))

Similarly, EBSCOhost platform APA PsychInfo, Scopus, Cochrane Library (wiley.com) were advance searched using the A-Z Database list.

TI Title: (student OR pediatric OR adolescen* OR “mind body” OR “physical activity” OR education) AND TI Title: (yoga OR qigong OR “tai chi” OR mindful OR walking OR movement OR physical OR intervention) TX All Text: (stress OR cortisol OR glucocortic* OR “blood pressure” OR “heart rate” OR “blood sugar” OR glucose OR stress OR experiment OR education OR primary OR secondary OR university OR tertiary OR graduate OR college)
